# Diagnosis and treatment of congenital tuberculosis: a systematic review of 92 cases

**DOI:** 10.1186/s13023-019-1101-x

**Published:** 2019-06-10

**Authors:** Chaofeng Li, Lili Liu, Yuhong Tao

**Affiliations:** 1 Department of Pediatrics, West China Second University Hospital, Sichuan University, No.20, Section 3, Renmin Nan Lu, Chengdu, 610041 Sichuan Province China; 2 Key Laboratory of Birth Defects and Related Diseases of Women and Children (Sichuan University), Ministry of Education, Chengdu, Sichuan China

**Keywords:** Tuberculosis, Congenital, Clinical analysis, Infant, Neonatal

## Abstract

**Background:**

Congenital tuberculosis is rare and carries a high mortality rate. Our objective was to summarize the current experience of the diagnosis and treatment of patients with congenital tuberculosis.

**Methods:**

In total, 73 reported cases of congenital tuberculosis published in Chinese and 19 patients with congenital tuberculosis admitted to West China Second University Hospital, Sichuan University, were retrospectively reviewed.

**Results:**

Sixty-four male and 28 female patients were identified. The majority of the patients were less than 3 weeks old at the time of presentation (range, 0–67 days). With regard to the tuberculosis type, 89 patients had pulmonary tuberculosis, and 20 patients had hepatic tuberculosis. There was active tuberculosis in 71 mothers, no tuberculosis in 12 mothers, and an unknown history of tuberculosis in 9 mothers. Fever, cyanosis, jaundice, shortness of breath, cough, pulmonary moist rales, hepatomegaly, splenomegaly and abdominal distention were the main clinical symptoms at the time of presentation. The abnormal ratios of chest, abdomen and head radiographic images were 97.53, 75 and 81.25%, respectively. The positive rates of acid-fast staining of sputum smears and tuberculosis bacillus DNA were 62.50 and 66.67%, respectively. The misdiagnosis rate was 59.78%. The overall mortality due to congenital tuberculosis was 43.48%. Respiratory failure was the most common cause of death. Sixty-five patients received anti-tuberculosis therapy, and of those, only 16 (15.38%) died.

**Conclusions:**

The clinical manifestations and radiographic findings of congenital tuberculosis are nonspecific. It is important to thoroughly evaluate the mothers of infants with suspected congenital tuberculosis. Good outcomes can be achieved in infants with the early identification of congenital tuberculosis and early administration of anti-tuberculosis treatment.

## Background

Congenital tuberculosis is an infection that develops as a result of an encounter between an infant and its mother with tuberculosis during the intrauterine period or during the normal birth process [1]. Tuberculous bacillemia during pregnancy may lead to infection of the placenta or the maternal genital tract, which may be transmitted to the fetus by hematogenous spread from the placenta to the umbilical vein or by the aspiration or ingestion of amniotic fluid contaminated by the placental or genital infection [2]. Hematogenous spread results in the formation of one or more primary complexes in the liver or lungs. On the other hand, the aspiration or ingestion of infected amniotic fluid results in primary complex formation in the lungs or gastrointestinal tract, respectively.

Tuberculosis is a major public health problem. According to the World Health Organization (WHO), one-third of the population is infected with tuberculosis, and 20 million people suffer from active tuberculosis [3]. However, congenital tuberculosis is rare. By 2005, fewer than 376 cases had been reported worldwide. China is a high-burden country with a high incidence of tuberculosis. Although the first case of congenital tuberculosis was reported in 1955 [4], only sporadic cases have been reported in China. To summarize the experience of the diagnosis and treatment of patients with congenital tuberculosis, a systematic review of 92 cases of congenital tuberculosis in China was conducted.

## Methods

### Sources

All Chinese publications that reported cases of congenital tuberculosis were assessed. A comprehensive search of the China National Knowledge Infrastructure (CNKI), Wanfang and Weipu databases was performed to identify all relevant papers published from 1976 to 2018 in China. The search keywords were congenital tuberculosis. The study language for all searches was limited to Chinese. A manual search was performed by checking the reference lists of the original reports and review articles that were retrieved through the electronic searches to identify studies not yet included in the computerized databases. In addition, patients with congenital tuberculosis admitted to the Department of Pediatrics, West China Second University hospital, Sichuan University from 1976 to 2018 were also included in this study.

According to the diagnostic criteria established by Cantwell in 1994 [5], the infant must have proved tuberculous lesions and at least one of the following: (1) lesions in the first week of life; (2) a primary hepatic complex or caseating hepatic granulomas; (3) tuberculous infection of the placenta or the maternal genital tract; or (4) exclusion of the possibility of postnatal transmission by a thorough investigation of contacts, including the infant's hospital attendants, and by adherence to existing recommendations for treating infants exposed to tuberculosis.

### Data collection

All potentially relevant cases were reviewed independently by the authors. We assessed the eligibility of each case with standardized data abstraction forms. Disagreements were resolved through discussion. All selected cases were evaluated for the main clinical characteristics. The following data were extracted from all eligible cases: sex, age at onset, age at diagnosis, maternal tuberculosis history, clinical manifestations, laboratory test results, imaging examination results, misdiagnoses, treatments and outcomes. According to the age at onset, the children were divided into the < 14-day-old group and the ≥14-day-old group. The clinical characteristics were compared between the two groups.

### Statistical analysis

All data were analyzed with IBM SPSS Statistics Version 20 (IBM Corp., Armonk, NY, USA). Normally distributed data are presented as the means ± standard deviation. Nonnormally distributed measurement data are expressed as medians. Count data are expressed as the constituent ratio. The measurement data were tested by *t*-tests, and the data were compared with the Chi-square test. The results were considered significant when *P* < 0.05.

## Results

### Demographic data

In total, 73 reported cases published in Chinese and 19 hospitalized patients with congenital tuberculosis in West China Second University Hospital, Sichuan University, were identified. Only 8 cases were reported in 1976–1990, 21 cases in 1991–2000, 36 cases in 2001–2010 and 21 cases in 2011–2018. The main demographic data from these cases are summarized in Table 1.

**Table 1:** Age and sex distribution of 92 infants with congenital tuberculosis

	Age at onset (days)					Age at diagnosis (days)					
Gender	≤7	8–14	15–21	22–28	≥29	≤21	22–42	43–63	64–84	≥85	Total
Male	13	16	16	8	11	16	22	14	5	6	127
Female	5	7	5	4	7	9	8	5	0	4	54
Total	18	23	21	12	18	25	30	19	5	10	181

Of the 92 cases, 64 were males, and 28 were females. There were 33 premature infants, 42 full-term infants and 17 infants with unknown gestational ages. The age at onset was within 3 weeks of birth in 63 cases (67.39%), with 18 (19.56%) within less than 7 days. The age at diagnosis ranged from 2 to 168 days. Only 3 infants (3.37%) were less than 7 days old. The time from onset to diagnosis ranged from 1 to 148 days, with 20 cases at less than 7 days, 25 cases at 8–14 days, 12 cases at 15–21 days, 4 cases at 22–28 days, and 23 cases at more than 29 days.

### Tuberculosis types in children and their mothers

Multiple organs were involved in congenital tuberculosis. Of the 92 cases, 89 cases were pulmonary tuberculosis (96.74%), 20 cases were hepatic tuberculosis (21.74%), 15 cases were lymph node tuberculosis (16.30%), 13 cases were tuberculous meningitis (14.13%), 11 cases were splenic tuberculosis (11.96%), 7 cases were renal tuberculosis (7.61%), 5 cases were adrenal tuberculosis (5.43%), 2 cases were thymic tuberculosis (2.17%), and 2 cases were tuberculous peritonitis (2.17%).

There were 71 mothers (77.18%) with histories of tuberculosis, 12 mothers (13.04%) without histories of tuberculosis and 9 mothers (9.78%) with unknown tuberculosis histories. Regarding the types of tuberculosis among the mothers, pulmonary tuberculosis occurred in 60 (84.51%), reproductive tuberculosis in 8 (11.27%), tuberculous meningitis in 6 (8.45%), tuberculous pleurisy in 5 (7.04%), tuberculous peritonitis in 3 (4.23%), placental tuberculosis in 1 (1.41%) and tuberculous pelvic inflammation in 1 (1.41%).

Seven mothers (9.86%) were diagnosed with tuberculosis before pregnancy, 16 (22.54%) were diagnosed during pregnancy and 48 (67.6%) were diagnosed after giving birth. During pregnancy, 21 mothers (22.83%) had clinical manifestations, 46 mothers (50%) had no clinical manifestations, and 25 mothers (27.17%) had unknown statuses regarding clinical manifestations.

### Main symptoms and signs

The main clinical manifestations were nonspecific and included fever, cyanosis, jaundice, shortness of breath, cough, pulmonary moist rales, hepatomegaly, splenomegaly and abdominal distention. As shown in Table 2, the majority of the clinical manifestations in the onset time < 14 days (41 cases) and onset time ≥ 14 days (51 cases) groups were similar. The incidence of jaundice in the onset time < 14 days group was higher than that in the onset time ≥ 14 days group (*P* < 0.05). The incidence of cough in the ≥14 days group was higher than that in the < 14 days group (*P* < 0.05).

**Table 2:** Main clinical manifestations in 92 infants with congenital tuberculosis

	Age at onset				
Clinical manifestations	≤14 days n/N (%)	> 14 days n/N (%)	Total	*χ* ^2^	*P*-value
Systemic manifestations					
Fever	31/41 (75.61)	43/51 (84.31)	74/92 (80.43)	1.094	0.296
Cyanosis	17/41 (41.46)	22/51 (43.14)	39/92 (42.39)	0.026	0.872
Jaundice	13/41 (31.71)	7/51 (13.73)	20/92 (21.74)	4.320	0.038
Pallor	7/41 (17.07)	10/51 (19.61)	17/92 (18.48)	0.097	0.756
Lymphadenopathy	4/41 (9.76)	4/51 (7.84)	8/92 (8.70)	0.000	1.000^1^
Respiratory system					
Respiratory distress	26/41 (63.41)	29/51 (56.86)	55/92 (59.78)	0.406	0.524
Cough	17/41 (41.46)	33/51 (64.71)	50/92 (54.35)	4.949	0.026
Pulmonary moist rales	12/41 (29.27)	20/51 (39.22)	32/92 (34.78)	0.991	0.319
Triple restriction	5/41 (12.20)	10/51 (19.61)	15/92 (16.30)	0.915	0.339
Foaming at the mouth	3/41 (7.32)	2/51 (3.92)	5/92 (5.43)	0.063	0.801^1^
Digestive system					
Hepatomegaly	28/41 (68.29)	35/51 (68.63)	63/92 (68.48)	0.001	0.973
Splenomegaly	18/41 (43.90)	26/51 (50.98)	44/92 (47.83)	0.456	0.499
Abdominal distention	10/41 (24.39)	11/51 (21.57)	21/92 (22.83)	0.103	0.749
Poor appetite	2/41 (4.88)	8/51 (15.69)	10/92 (10.87)	1.738	0.187^1^
Poor feeding	4/41 (9.76)	5/51 (9.80)	9/92 (9.78)	0.000	1.000^1^
Vomiting	2/41 (4.88)	4/51 (7.84)	6/92 (6.52)	0.022	0.883^1^
Diarrhea	2/41 (4.88)	4/51 (7.84)	6/92 (6.52)	0.022	0.883^1^
Nervous system					
Mental fatigue	6/41 (14.63)	11/51 (21.57)	17/92 (18.48)	0.726	0.394
Seizures	5/41 (12.20)	5/51 (9.80)	10/92 (10.87)	0.001	0.977^1^
Skin	7/41 (17.07)	3/51 (5.88)	10/92 (10.87)	1.896	0.168^1^

^1^Continuity correction

### Main laboratory findings

Laboratory test findings were nonspecific. As shown in Table 3, the positive rates of acid-fast staining of sputum smears and tuberculosis bacillus DNA detected by polymerase chain reaction (PCR) were 62.5 and 66.67%, respectively. However, the rates of increased erythrocyte sedimentation rate (ESR), positive purified protein derivative (PPD) test and positive tuberculosis bacillus culture were low.

**Table 3:** Laboratory examination results in 92 infants with congenital tuberculosis

Examination	No. of patients tested	No. positive	Percentage (%)
Etiology			
PPD test	31	7	22.58
Acid-fast staining of gastric juice smears	28	14	50.00
Culture of tuberculosis bacilli in gastric juice	4	1	25.00
Acid-fast staining of sputum smears	24	15	62.50
Culture of tuberculosis bacilli in sputum	20	1	5.00
Acid-fast staining of cerebrospinal fluid smears	12	1	8.33
Culture of tuberculosis bacilli in cerebrospinal fluid	10	0	0
Detection of tuberculosis DNA in sputum by PCR	18	12	66.67
Leukocyte count > 12 × 10^9^/L	74	53	71.62
Neutrophil > 50%	65	47	72.31
Hemoglobin< 110 g/L	65	34	52.31
Platelet count< 100 × 10^9^/L	50	11	22.00
C-reactive protein> 10 mg/L	38	25	65.79
Erythrocyte sedimentation rate > 20 mm/h	22	8	36.36
Serum alanine aminotransferase level > 40 U/L	50	15	30.00
Serum aspartate transferase level > 40 U/L	46	27	58.7
Serum total protein < 60 g/L	30	15	50.00
Serum albumin level < 35 g/L	32	20	62.50
Serum globin level < 25 g/L	31	11	35.48
Serum total bilirubin> 20 μmol/L	42	24	57.14
Serum direct bilirubin level > 7 μmol/L	34	18	52.94
Serum lactate dehydrogenase level > 250 U/L	20	20	100
Serum γ-glutamyl transpeptidase level > 50 U/L	22	14	63.64
Serum creatinine level > 40 μmol/L	22	4	18.18

### Main radiographic imaging results

In total, 81 patients (88.04%) underwent thoracic X-ray imaging examination. Of the 81 patients, 79 (97.53%) had abnormal findings, including diffuse miliary nodules in 29 (35.44%), diffuse pneumonia-like changes in 28 (35.89%), hilar lymph node tuberculosis in 12 (15.19%), bronchopneumonia in 7 (8.86%), pleural effusion in 5 (6.33%), hematogenous disseminated pulmonary tuberculosis in 3 (3.80%), primary pulmonary syndrome in 1 (1.27%) and lipoid aspiration pneumonia with interstitial emphysema in 1 (1.27%).

Thirty-two patients (34.78%) underwent abdominal X-ray imaging examination. Of the 32 patients, 24 (75%) had abnormal findings, including hepatomegaly in 17 (70.83%), splenomegaly in 14 (58.33%), ascites in 6 (25%), hilar lymphadenopathy in 3 (12.5%) and hilar lymphadenopathy in 2 (8.33%).

Sixteen patients (17.39%) underwent cranial X-ray imaging examination. Of the 16 patients, 13 (81.25%) had abnormal findings, including brain parenchymal density reduction in 9 (69.24%), intracranial hemorrhage in 1 (7.69%), brain atrophy in 1 (7.69%), midbrain and right cerebellar nodule shadow in 1 (7.69%) and encephalomalacia in 1 (7.69%).

### Misdiagnoses

Fifty-five patients (59.78%) were misdiagnosed. The misdiagnoses included neonatal pneumonia in 49 patients (89.09%), neonatal sepsis in 18 patients (32.73%), neonatal hepatitis syndrome in 3 patients (5.45%) and anemia in 1 patient (1.82%).

### Treatment and outcomes

Forty patients (43.48%) died. The time of death ranged from 7 to 168 days (median 36.5 days). Among the 40 children who died, the causes of death included respiratory failure in 26 (65%), multiple organ dysfunction syndrome in 3 (7.5%), intracranial hemorrhage in 1 (2.5%) and unknown causes in 10 (25%). After definite diagnosis, 65 patients received anti-tuberculosis therapy. Eight patients were cured, 39 patients improved, and 16 patients (15.38%) died.

## Discussion

Congenital tuberculosis has a high mortality rate. In the present study, the mortality rate was 43.48%. The most common cause of death was respiratory failure. This is consistent with the results of Abughali et al. [6] and Abalain et al. [7]. The causes of the high mortality rate are as follows: (1) lack of awareness and delayed diagnosis and (2) rapid progress and delayed treatment.

It is still challenging to distinguish congenital from postnatally acquired tuberculosis. The age at the onset of congenital tuberculosis is not uniform. Infants with congenital tuberculosis may be symptomatic at birth, but symptoms can also occur within days to weeks after birth. Due to the different immune status of every newborn, the onset of the disease is slower in some children [8]. Congenital tuberculosis should still be diagnosed if it is clear that the tuberculosis in the infant originated from the mother before birth or at birth. Sen et al. [9] reported that the symptoms of congenital tuberculosis mainly occur within 3 weeks after birth, at an average age of 28 days. Cantwell et al. [5] reported that the average age at the onset of congenital tuberculosis was 24 days (range, 1–84 days). Vogel et al. [10] reported that some patients with congenital tuberculosis did not develop symptoms until 3 months after birth or longer. The longest duration between birth and the onset of symptoms was 154 days. Schaaf et al. [11] reported that the oldest onset age of a patient with congenital tuberculosis was 112 days after birth. In the present study, the onset age of patients with congenital tuberculosis ranged from 0 to 67 days. In total, 67.39% of patients developed symptoms within 3 weeks of birth, and patients who developed symptoms in fewer than 7 days accounted for 19.56% of all patients with congenital tuberculosis. In addition, disseminated Bacilli Calmette Guerin (BCG) disease should be excluded [12]. Because BCG vaccination is completed within 24 h of birth in China, the onset time for disseminated BCG disease may overlap with that of congenital tuberculosis. Patients with disseminated BCG disease have clear vaccination histories, and most have local manifestations at vaccination sites and a prominent tendency towards lymph node involvement.

Due to the lack of a host immune response, congenital tuberculosis is essentially systemic disseminated tuberculosis. In the present study, multiple organs were involved, especially the lung. However, hepatic tuberculosis, lymph node tuberculosis, tubercular meningitis, splenic tuberculosis, renal tuberculosis, adrenal tuberculosis, thymic tuberculosis, pleural tuberculosis and tubercular peritonitis have also been found. The most common manifestations of congenital tuberculosis are poor appetite, fever, irritability, hypoplasia, weight loss, cough, respiratory distress, hepatosplenomegaly, splenomegaly, lymphadenopathy and abdominal distention [13]. Severe manifestations include meningitis, septicemia, miliary tuberculosis, unremitting or recurrent pneumonia, and disseminated intravascular coagulation [14]. However, these clinical manifestations are similar to those of other infectious diseases, such as bacterial pneumonia, sepsis, purulent meningitis, and infantile hepatitis syndrome [7]. Congenital tuberculosis is often misdiagnosed before birth or even found during autopsy. In the present study, there were no significant differences in most of the clinical manifestations between the onset age < 14 days group and the onset age ≥ 14 days group. The incidence of cough in the onset age ≥ 14 days group was significantly higher than that in the onset age < 14 days group. However, whether the onset age was ≤14 days or>14 days, lung moist rales were uncommon (29.27 and 39.22%, respectively). After excluding fungal infections, congenital tuberculosis should be considered in infants younger than 2 months of age presenting with fever, pneumonia, hepatosplenomegaly or unexplained sepsis. In particular, if various antibiotic treatments have been demonstrated to be ineffective and the patient’s condition deteriorates after a high dose of glucocorticoid steroids, congenital tuberculosis should be considered.

Pregnancy may present a state of physiological immunosuppression. The risk of tuberculosis in pregnancy is probably also increased, leading to an increased risk of congenital tuberculosis [15]. In countries with high tuberculosis burdens, the prevalence of active tuberculosis among pregnant women and postpartum women is as high as 60 cases/100,000 population per year [16]. Therefore, routine screening for potential or active tuberculosis infections during pregnancy is critical [17, 18]. Pregnant mothers may have systemic disseminated tuberculosis or genital tuberculosis (endometrial, cervical or placental tuberculosis). Among these types of tuberculosis, maternal genital tuberculosis is the most likely to lead to congenital tuberculosis. Peng et al. [19] showed that 162 mothers of 170 infants with congenital tuberculosis had active tuberculosis throughout pregnancy or the postpartum period; however, 121 mothers had no history of tuberculosis before pregnancy and were diagnosed during pregnancy. In the present study, 77.18% of the pregnant mothers had an antenatal history of tuberculosis, and 13.04% had no history of tuberculosis. The types of tuberculosis included pulmonary tuberculosis (84.51%), reproductive tuberculosis (11.27%) and placental tuberculosis (1 mother). However, only 22.83% of the mothers had clinical manifestations during pregnancy, while 50% of the mothers had no clinical manifestations, and 67.6% of the mothers were diagnosed after giving birth. Cantwell et al. [5] found that 60–70% of mothers of patients had no symptoms. Most mothers are diagnosed with tuberculosis only after the child has been diagnosed with tuberculosis. Espiritu et al. [15] reviewed 32 cases of congenital tuberculosis, and 24 of the pregnant mothers were asymptomatic. Lee et al. [20] believed that morphological and histological examination of the placenta during childbirth was very useful for achieving a definite diagnosis. In many cases, the mothers had subclinical tuberculosis, which was confirmed only after the disease was diagnosed in the infants. Therefore, it is very important to thoroughly evaluate the mothers of infants with suspected congenital tuberculosis. If the pregnant mothers have active tuberculosis during pregnancy, newborns should be checked for congenital tuberculosis whether or not they have symptoms after birth.

Identifying the presence of tuberculosis bacilli by body fluid cultures, acid-fast staining or tissue biopsy is the gold standard for the diagnosis of tuberculosis. After the exclusion of postnatally acquired tuberculosis, congenital tuberculosis can be diagnosed. The demonstration of a hepatic primary complex usually requires an open surgical procedure or autopsy to confirm liver and regional lymph node involvement. Although the sensitivity of liver biopsy for the diagnosis of congenital tuberculosis is 100% [21], liver biopsy is rarely employed because it is invasive. In the present study, the results of the traditional tuberculosis test in most children are normal at the time of diagnosis, with no evidence of an increased ESR and a negative PPD test. The immune system of neonates is not well developed, and no allergic reactions occur within 2 to 10 weeks after the latest infection; the PPD test can be negative even in patients with severe tuberculosis. Even if the PPD test is negative, the disease cannot be ruled out. Previous research also suggested that gastric or tracheal aspirates were positive in 80% of children with congenital tuberculosis [22]. The detection of *Mycobacterium tuberculosis* DNA in bronchoalveolar lavage fluid by PCR is also an effective method for the diagnosis of tuberculosis [23]. In the present study, the positive rates of acid-fast staining in sputum or gastric juice and PCR amplification of tuberculosis bacilli DNA in sputum samples were high, but the positive rates of tuberculosis bacilli cultures from sputum or gastric juice samples and the PPD test was still low. Therefore, repeated acid-fast staining in sputum or gastric juice samples or tuberculosis bacillus DNA detection in bronchoalveolar lavage fluid are the techniques recommended to improve the positive rate of diagnosis. Nontuberculosis mycobacterium (NTM) infection should also be excluded in the diagnosis of congenital tuberculosis. NTM infection in young children is rare, and congenital NTM infection is exceedingly rare. The diagnostic basis of NTM is still bacteriological.

In the present study, 97.53% of patients had abnormal chest imaging findings. In addition, diffuse miliary nodules (35.44%) and diffuse pneumonia-like changes (35.89%) were very common. Peng et al. [19] reported that 44.68% of the chest radiographs of patients with congenital tuberculosis showed nodular changes, which was similar to the result in the present study. Although most children with congenital tuberculosis have abnormal chest X-ray images, it is not a definite diagnostic method. Ultrasonography is also very useful in the diagnosis of liver, spleen and kidney lesions. Patients suspected of having congenital tuberculosis should be routinely examined with abdominal CT. Once the primary focus of tuberculosis is found in the liver, the diagnosis can be confirmed. In the present study, 75% of imaging findings were abnormal, and hepatomegaly and splenomegaly were the most commonly observed abnormalities. This is similar to the results of Raj et al. [24], Lee et al. [20] and Peng et al. [19]. It should be noted that animal experiments show that typical imaging changes usually occur 2 to 3 weeks after the initial tuberculosis infection. In the present study, 2.47% of thoracic images and 25% of abdominal images were still normal. Therefore, it is necessary to dynamically monitor the changes in the imaging results of patients. Negative initial imaging cannot exclude the disease.

Patients with congenital tuberculosis and those with postnatally acquired disease should be treated similarly [19]. Infants should receive isoniazid (10–15 mg/kg.d), rifampin (10–20 mg/kg.d), pyrazinamide (15–30 mg/kg.d), and either streptomycin (20–30 mg/kg.d) or ethambutol (15–25 mg/kg.d) for the first 2 months, followed by isoniazid and rifampin for 4–10 months, depending on the severity of the disease [5]. In 2007, the WHO reported that the rates of treatment failure and mortality have increased due to the emergence of multidrug-resistant tuberculosis strains [18]. In the present study, the mortality rate of congenital tuberculosis was only 15.38% after diagnosis and anti-tuberculosis treatment. Therefore, early treatment may improve the prognosis.

Of course, there were some limitations in the present study. The cases come from the literature reported in Chinese. The quality of the studies varied; some data were incomplete, the time span was large, and follow-up could not be performed.

## Conclusions

Congenital tuberculosis is nonspecific with regard to the clinical manifestations and radiographic findings. The misdiagnosis and mortality rates are high, and the prognosis is poor. It is vital to thoroughly investigate the antenatal history of tuberculosis in the pregnant mother to confirm the diagnosis of congenital tuberculosis. Early diagnosis and treatment are essential to improve the prognosis of the disease.

## Data Availability

Data sharing not applicable to this article as no datasets were generated or analysed during the current study.

## Abbreviations

BCG: Bacilli Calmette Guerin
CNKI: China National Knowledge Infrastructure
ESR: Erythrocyte sedimentation rate
NTM: Nontuberculosis mycobacterium
PCR: Polymerase chain reaction
PPD: Purified protein derivative
WHO: World Health Organization
